# Reliability of the Evidence to Guide Decision-Making in Acupuncture for Functional Dyspepsia

**DOI:** 10.3389/fpubh.2022.842096

**Published:** 2022-04-01

**Authors:** Jinke Huang, Jiali Liu, Zhihong Liu, Jing Ma, Jinxin Ma, Mi Lv, Fengyun Wang, Xudong Tang

**Affiliations:** ^1^Department of Gastroenterology, Xiyuan Hospital, China Academy of Chinese Medical Sciences, Beijing, China; ^2^Department of Gastroenterology, Peking University Traditional Chinese Medicine Clinical Medical School (Xiyuan), Beijing, China; ^3^China Academy of Chinese Medical Sciences, Beijing, China

**Keywords:** reliability, evidence, decision-making, acupuncture, functional dyspepsia

## Abstract

**Background and Aims:**

There has been a significant increase in the number of systematic reviews (SRs)/meta-analyses (MAs) investigating the effects of acupuncture for functional dyspepsia (FD). To systematically collate, appraise, and synthesize the current evidence, we carried out an umbrella review of SRs/MAs.

**Methods:**

Systemic reviews/meta-analyses on acupuncture for FD were collected by searching major medical databases. The included studies were evaluated in terms of methodological quality, reporting quality, and evidence quality using the criteria from the Assessment of Multiple Systematic Reviews 2 (AMSTAR-2) tool, the Preferred Reporting Items for Systematic Reviews and Meta-Analyses (PRISMA) statement, and the Grades of Recommendation, Assessment, Development, and Evaluation (GRADE) system, respectively.

**Results:**

Ten SRs/MAs were analyzed for this study. The methodological quality, reporting quality, and evidence quality of the included SRs/MAs were generally unsatisfactory. Lack of protocol registration, no list of excluded trials, or lack of a comprehensive search strategy were the main limitations. No high-quality evidence was found to support the effects of acupuncture for FD; the qualitative data synthesis relied on low quality trials with small sample sizes and was the main factor for evidence degradation.

**Conclusions:**

Acupuncture seems to have a promising efficacy in the treatment of FD. It provides a new and prospective therapeutic method for FD. Although the quality of the included SRs/MAs was generally low and defects were frequent, this umbrella review highlights areas where improvement in methodology is required.

## Introduction

Functional dyspepsia (FD) is a functional gastrointestinal disease marked chiefly by early satiation, postprandial fullness, epigastric pain, and epigastric burning (1). The annual prevalence of FD is 9–10%, with 15% of patients having frequent (>3 attacks per week) and chronic (>3 months per year) symptoms (2). As a result of continuous medical treatment and continuous drug use, indigestion symptoms create a huge socio-economic burden and have a negative impact on the quality of life (3, 4). It is reported that the total yearly direct cost of presentation and treatment is $805 per patient, yet the economic losses of FD on patients are much higher in combination with the indirect costs caused by resignation or reduced productivity (4). Abnormal gastric motility (delayed gastric emptying), impaired gastric adaptability to food, and visceral hypersensitivity (gastric hyperdistention sensitivity and acid hypersensitivity) are usually considered to be the main pathophysiological mechanisms of FD (5). Current pharmacological options include antidepressants, antacids, anxiolytics, prokinetics, and *Helicobacter pylori* eradication (6). However, none of the available therapies are effective in the majority of patients (6).

Acupuncture has been widely used to treat functional gastrointestinal disorders including FD. A literature search yielded that an increasing number of systematic reviews (SRs)/meta-analyses (MAs) regarding this topic have been conducted. However, not all SRs/MAs can provide reliable evidence, and low-quality SRs/MAs may instead mislead decision-makers (7). Furthermore, in an attempt to ensure validity of evidence and improve the quality of SRs/MAs, the measurement tools like the Assessment of Multiple Systematic Reviews 2 (AMSTAR-2) (8), the Preferred Reporting Items for Systematic Reviews and Meta-Analyses (PRISMA) (9), and the Grades of Recommendation, Assessment, Development, and Evaluation (GRADE) (10) were published in 2007, 2009, and 2004, respectively. An umbrella review is defined as an evidence-based medicine approach to the systematic review of all SRs/MAs on the same topic. Conducting an umbrella review helps to integrate the evidence more comprehensively, thereby providing higher quality evidence for clinicians. Hence, to systematically collate, evaluate, and synthesize current evidence on acupuncture for FD, we conducted this study.

## Methods

The protocol of this study was registrated in PROSPERO registry (CRD42021262020), and the methodology was performed following the criteria of the Cochrane Handbook.

### Criteria for Considering Studies

The criteria for selection were as follows: (a) SRs/MAs of only enrolled randomized controlled trials (RCTs), (b) subjects were diagnosed with FD according to the Rome diagnostic criteria, (c) interventions applied to the experimental group included acupuncture alone or in combination with conventional medicine (CM), while interventions applied to the control included sham acupuncture or CM, (d) reporting at least one prespecified outcome: symptom score (SS), nepean dyspepsia index (NDI), main frequency of electrogastrogram (MF-EGG), gastric half-emptying time (GHET), plasma motilin level (PML), and effective rate (ER). The exclusion criteria were as follows: (a) repeated publications, (b) graduate dissertation, (c) lack of further data, and (d) conference abstracts.

### Search Strategy

Systematic reviews/Meta-analysis of acupuncture on FD were systematically searched in the following databases from their inception till August 2021: the Cochrane Library, PubMed, Web of Science, Embase, Chinese Biomedical Database, Chinese VIP information, and Wanfang Data. Functional dyspepsia, acupuncture, and systematic review were used as key search terms. A strategy for using PubMed search is shown in Table 1.

**Table 1:** Detailed retrieval strategy for PubMed.

**Query**	**Search term**
#1	Dyspepsia[Mesh]
#2	Dyspepsia*[Title/Abstract] OR Functional dyspepsia* [Title/Abstract] OR Indigestion* [Title/Abstract] OR non-ulcer dyspepsia* [Title/Abstract]
#3	#1 OR #2
#4	Acupuncture [Mesh]
#5	Acupuncture [Title/Abstract] OR pharmacoacupuncture[Title/Abstract] OR acupotomy[Title/Abstract] OR acupotomies[Title/Abstract] OR pharmacopuncture[Title/Abstract] OR needle[Title/Abstract] OR needling[Title/Abstract] OR dry-needling[Title/Abstract] OR body-acupuncture[Title/Abstract] OR electroacupuncture[Title/Abstract] OR electro-acupuncture[Title/Abstract] OR auricular acupuncture[Title/Abstract] OR warm needle[Title/Abstract]
#6	#4 OR #5
#7	Meta-Analysis as Topic [Mesh]
#8	Systematic review[Title/Abstract] OR Meta-Analysis[Title/Abstract] OR meta analysis[Title/Abstract] OR meta-analyses[Title/Abstract] OR metaanalysis[Title/Abstract]
#9	#7 OR #8
#10	#3 AND #6 AND #9

### Literature Screening and Data Extraction

Literature selection and data extraction were conducted by two independent reviewers. For literature selection, titles and abstracts were screened first, and the full texts of potential literature were read for further evaluation. From the articles included, the following data were extracted: first author, publication year, sample size, interventions, outcomes, and main results. Disagreements were settled *via* discussion and consensus.

### Quality Assessment

Methodological quality, reporting quality, and evidence quality of the enrolled studies were evaluated by two independent reviewers using the AMSTAR-2 tool, the PRISMA statement, and the GRADE system, respectively. The AMSTAR-2 consisted of 16 items, and the methodological quality was ranked as high, moderate, low, or critically low (8). The PRISMA statement consisted of 27 items, with each of the 16 items given a rating of yes, no, or partly yes (9). Evidence quality with GRADE was considered from five aspects (risk of bias, inconsistency, indirectness, imprecision, and publication bias) and given a rating of high to critically low (10). Disagreements were settled *via* discussion and consensus.

## Results

### Literature Screening

**Figure 1:**
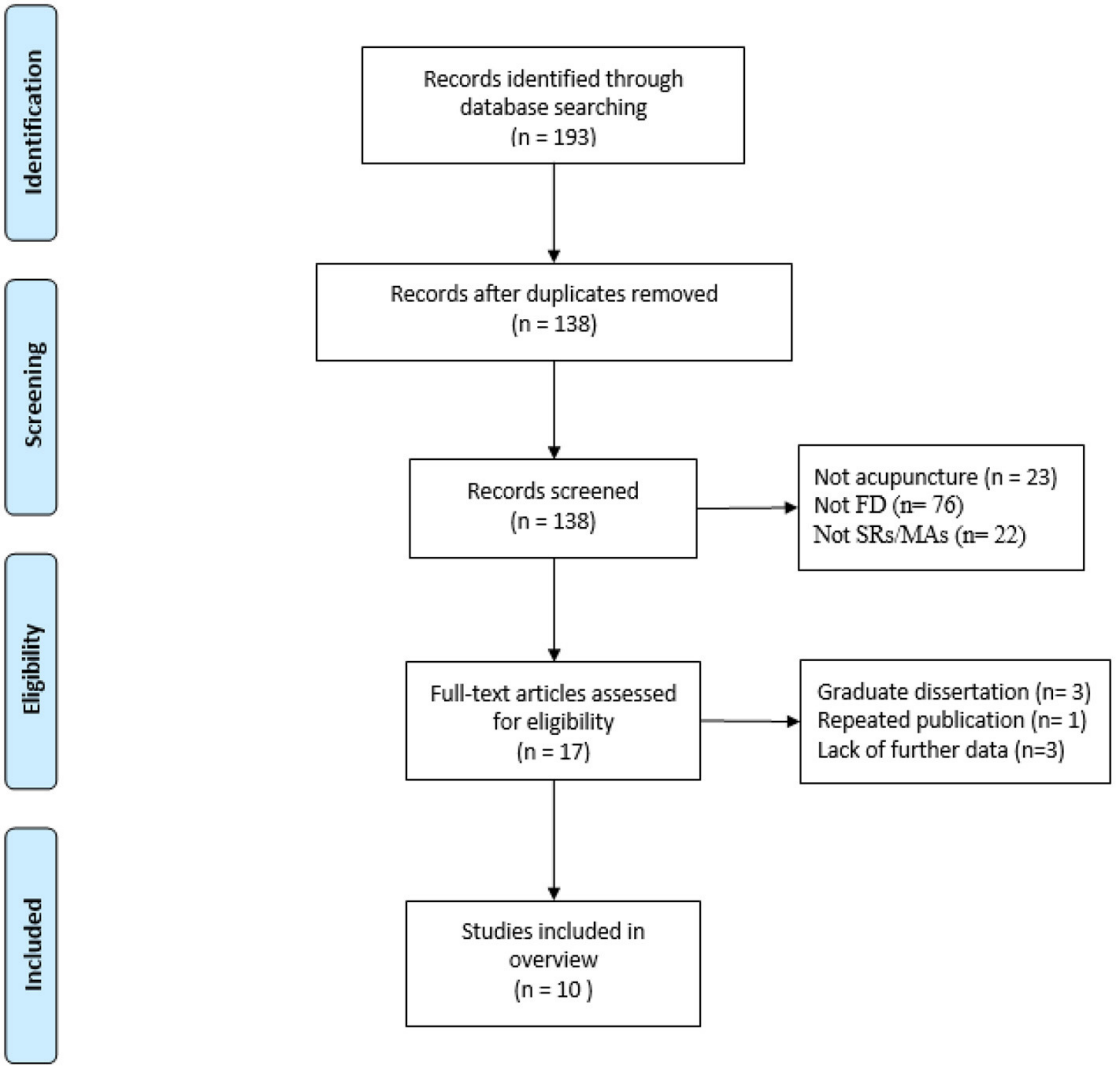
Literature screening flowchart.

The literature search yielded 193 citations, of which 55 duplicates were removed. After screening the title and abstract, 121 citations were excluded and the remaining 17 were further evaluated through full-text reading. Finally, 10 SRs/MAs (11–20) met the inclusion criteria (Figure 1).

### Characteristics of the Primary Studies

Then, SRs/MAs published from 2014 to 2021 were included. The interventions applied to the experimental groups were acupuncture alone or in combination with CM, while those administered to the control groups were CM or sham acupuncture. More details are presented in Table 2.

**Table 2:** A description of the characteristics.

**References**	**Country**	**Trials (subjects)**	**Experimental intervention**	**Control intervention**	**Quality assessment**	**Meta-analyses**	**Results summary**
Han (11)	China	10(1,202)	AT	Sham AT, CM	Cochrane criteria	Yes	Regarding the improvement in the symptoms of FD, the effect of acupuncture was superior to the sham-acupuncture group or the western medication group.
Mao (12)	China	7(853)	AT	Sham AT, CM	Cochrane criteria	Yes	Acupuncture was more effective than placebo in treating FD, while it was comparable to CM.
Yuan (13)	China	31(2,571)	AT	CM	Cochrane criteria	Yes	Compared with CM, acupuncture can significantly improve the effective rate, functional dyspeptic symptoms and motilin levels.
Zhou (14)	China	24(3,097)	AT, AT+CM	Sham AT, CM	Cochrane criteria	Yes	Acupuncture can effectively alleviate the symptoms of FD and improve the quality of life of patients.
Pang (15)	China	16(1,436)	AT	Sham AT, CM	Cochrane criteria	Yes	Acupuncture was superior to sham acupuncture and CM in improving symptoms and quality of life in FD patients.
Kim (16)	Korea	20(1,423)	AT, AT+CM	Sham AT, CM	Cochrane criteria	Yes	Acupuncture was superior to sham acupuncture and CM in improving symptoms and quality of life in FD patients.
Lan (17)	China	7(542)	AT	Sham AT, CM	Cochrane criteria	Yes	Whether acupuncture was more effective or safer than other treatments for the treatment of FD remains inconclusive.
Peng (18)	China	7(1,044)	AT	CM	Cochrane criteria	Yes	The therapeutic effect of acupuncture on FD was equivalent to that of CM.
Jin (19)	China	20(1,301)	AT	CM	Jadad	Yes	Acupuncture was more effective than CM in the treatment of FD, and had a lower risk of adverse events.
Wu (20)	China	16(1,088)	AT	CM	Jadad	Yes	Acupuncture was more effective than CM in the treatment of FD, and had a lower risk of adverse events.

### Methodological Quality

**Figure 2:**
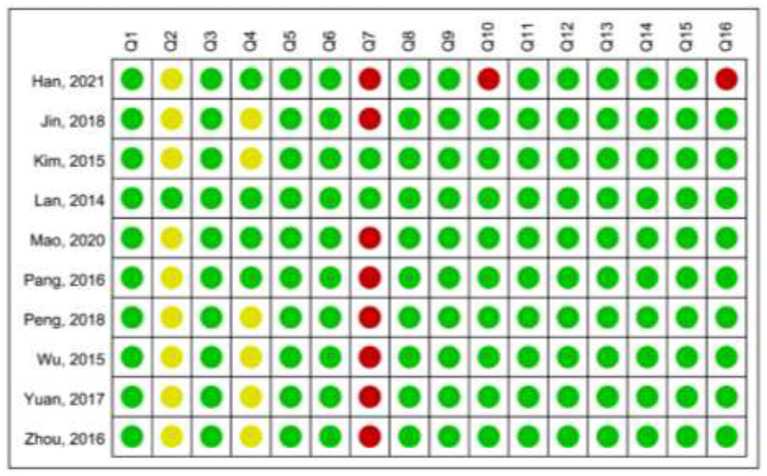
Summary of the Assessment of Multiple Systematic Reviews 2 (AMSTAR-2).

**Figure 3:**
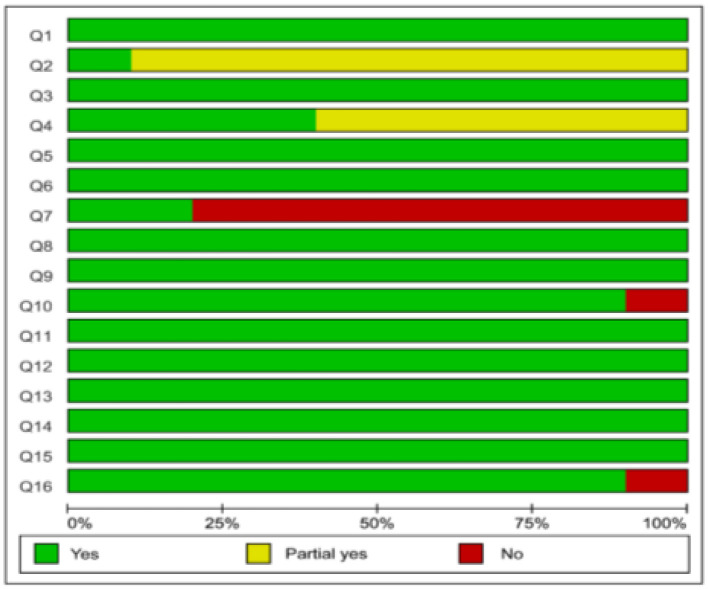
Graphical representation of the AMSTAR-2.

According to the AMSTAR-2, only one Cochrane SR/MA presented high level of methodological quality, while the other SRs/MAs presented critically low levels of methodological quality. Lack of protocol registration, no list of excluded trials, or lack of a comprehensive search strategy were the main limitations of the included SRs/MAs (Figures 2, 3).

### Reporting Quality

According to the PRISMA statement, only one Cochrane SR/MA fully reported information on the 27 items, while the other SRs/MAs missed serious information in item Q5 (protocol and registration), Q8 (search), and Q27 (funding). More details are shown in Table 3.

**Table 3:** Result of the PRISMA checklist.

**Section/Topic**	**Items**	**(11)**	**(12)**	**(13)**	**(14)**	**(15)**	**(16)**	**(17)**	**(18)**	**(19)**	**(20)**	**Compliance (%)**
Title	Q1. Title	Y	Y	Y	Y	Y	Y	Y	Y	Y	Y	100%
Abstract	Q2. Structured summary	Y	Y	Y	Y	Y	Y	Y	Y	Y	Y	100%
Introduction	Q3. Rationale	Y	Y	Y	Y	Y	Y	Y	Y	Y	Y	100%
	Q4. Objectives	Y	Y	Y	Y	Y	Y	Y	Y	Y	Y	100%
Methods	Q5. Protocol and registration	N	N	N	N	N	N	Y	N	N	N	10%
	Q6. Eligibility criteria	Y	Y	Y	Y	Y	Y	Y	Y	Y	Y	100%
	Q7. Information sources	Y	Y	Y	Y	Y	Y	Y	Y	Y	Y	100%
	Q8. Search	Y	Y	PY	PY	Y	PY	Y	PY	PY	PY	40%
	Q9. Study selection	Y	Y	Y	Y	Y	Y	Y	Y	Y	Y	100%
	Q10. Data collection process	Y	Y	Y	Y	Y	Y	Y	Y	Y	Y	100%
	Q11. Data items	Y	Y	Y	Y	Y	Y	Y	Y	Y	Y	100%
	Q12. Risk of bias in individual studies	Y	Y	Y	Y	Y	Y	Y	Y	Y	Y	100%
	Q13. Summary measures	Y	Y	Y	Y	Y	Y	Y	Y	Y	Y	100%
	Q14. Synthesis of results	Y	Y	Y	Y	Y	Y	Y	Y	Y	Y	100%
	Q15. Risk of bias across studies	Y	Y	Y	Y	Y	Y	Y	Y	Y	Y	100%
	Q16. Additional analyses	Y	Y	Y	Y	Y	Y	Y	Y	Y	Y	100%
Results	Q17. Study selection	Y	Y	Y	Y	Y	Y	Y	Y	Y	Y	100%
	Q18. Study characteristics	Y	Y	Y	Y	Y	Y	Y	Y	Y	Y	100%
	Q19. Risk of bias within studies	Y	Y	Y	Y	Y	Y	Y	Y	Y	Y	100%
	Q20. Results of individual studies	Y	Y	Y	Y	Y	Y	Y	Y	Y	Y	100%
	Q21. Synthesis of results	Y	Y	Y	Y	Y	Y	Y	Y	Y	Y	100%
	Q22. Risk of bias across studies	Y	Y	Y	Y	Y	Y	Y	Y	Y	Y	100%
	Q23. Additional analysis	Y	Y	Y	Y	Y	Y	Y	Y	Y	Y	100%
Discussion	Q24. Summary of evidence	Y	Y	Y	Y	Y	Y	Y	Y	Y	Y	100%
	Q25. Limitations	Y	Y	Y	Y	Y	Y	Y	Y	Y	Y	100%
	Q26. Conclusions	Y	Y	Y	Y	Y	Y	Y	Y	Y	Y	100%
Funding	Q27. Funding	N	Y	Y	Y	Y	Y	Y	Y	Y	N	80%

### Evidence Quality

With the GRADE system, the evidence quality of all concerned outcome indicators ranged from “critically low” to “moderate” due to the risk of bias, inconsistency, imprecision, and publication bias. Furthermore, upgrading factors such as large effect and evidence of dose-response gradient were not applicable to the included outcome indicators. More details are shown in Table 4.

**Table 4:** Results of evidence quality.

**References**	**Treatments**	**Outcomes**	**Limitations**	**Inconsistency**	**Indirectness**	**Imprecision**	**Publication bias**	**Relative effect (95% CI)**	**Quality**
Han (11)	AT vs. Sham AT	SS	−1	0	0	−1	−1	SMD −3.03 (−3.56, −2.50)	CL
		ER	−1	0	0	−1	−1	OR 5.09 (3.30, 7.86)	CL
		MF-ECG	−1	0	0	−1	−1	MD −14.36 (−18.31, −10.41)	CL
	AT vs. CM	SS	−1	0	0	−1	−1	MD −3.03 (−3.56, −2.50)	CL
		ER	−1	0	0	−1	−1	OR 1.33 (0.70, 2.52)	CL
		MF-ECG	−1	0	0	−1	−1	MD 0.01 (−0.03, 0.01)	CL
		PML	−1	0	0	−1	−1	SMD −0.06 (−0.23, 0.11)	CL
		GHMT	−1	0	0	−1	−1	MD 0.23 (−1.94, 2.40)	CL
Mao (12)	AT vs. Sham AT	SS	−1	0	0	−1	−1	MD −3.44 (−4.21, −2.67)	CL
		MF-ECG	−1	−1	0	−1	−1	SMD −0.69 (−3.02, 4.40)	CL
	AT vs. CM	SS	−1	−1	0	0	−1	SMD −0.18 (−0.51, 0.16)	CL
		ER	−1	0	0	0	0	RR 1.04 (0.96,1.13)	M
		PML	−1	0	0	0	0	SMD 0.93 (−0.30, 1.55)	M
		GHMT	−1	0	0	−1	−1	SMD 0.02 (−0.16, 0.20)	CL
Yuan (13)	AT vs. CM	ER	−1	0	0	0	−1	OR 3.00 (2.33, 3.87)	L
		SS	−1	−1	0	0	0	WMD −1.21 (−2.13, −0.30)	L
		PML	−1	0	0	0	0	WMD 13.99 (0.45, 27.54)	M
Zhou (14)	AT vs. Sham AT	SS	−1	−1	0	0	0	SMD −1.23 (−2.00,−0.47)	L
	AT vs. CM	SS	−1	−1	0	0	0	SMD −0.30 (−0.77,0.16)	L
		PML	−1	−1	0	0	0	SMD 0.67 (−0.07,1.42)	L
Pang (15)	AT vs. Sham AT	NDI	−1	−1	0	−1	−1	MD 20.91 (6.55,35.26)	CL
Kim (16)	AT vs. Sham AT	SS	−1	0	0	−1	−1	MD 0.54 (0.18, 0.90)	CL
		ER	−1	−1	0	−1	−1	RR 2.66 (1.85, 3.82)	CL
	AT vs. CM	SS	−1	−1	0	0	0	MD 0.54 (0.33,0.76)	L
		ER	−1	−1	0	−1	−1	RR 1.18 (1.09, 1.27)	CL
Lan (17)	AT vs. CM	ER	−1	0	0	−1	−1	RR 1.02 (0.91,1.16)	CL
Peng (18)	AT vs. CM	ER	−1	0	0	0	0	RR 1.17 (1.10,1.24)	M
Jin (19)	AT vs. CM	ER	−1	−1	0	0	0	RR 1.21 (1.15,1.27)	L
		SS	−1	−1	0	−1	−1	MD −2.10 (−3.61,−0.59)	CL
		NDI	−1	0	0	−1	−1	MD 9.94 (7.93,11.94)	CL
Wu (20)	AT vs. CM	ER	−1	0	0	0	0	RR 1.18 (1.11,1.24)	M

*SS, symptom score; ER, effective rate; MF-EGG, main frequency of electrogastrogram; PML, plasma motilin level; GHMT, gastric half-emptying time; NDI, nepean dyspepsia index. CL, critically low; L, Low; M, moderate*.

### Meta-Analyses Outcomes of Intervention

#### Acupuncture vs. Sham Acupuncture

Five studies (11, 12, 14–16) compared the effects of acupuncture with sham acupuncture. Three studies (11, 12, 14, 16) reported significant improvements in SSs in the acupuncture group compared to the sham acupuncture. Two studies reported ERs (11, 16), and the meta-analysis results revealed that the acupuncture group was significantly superior to the sham acupuncture group. The MF- EGG was reported in two studies (11, 12). One study (11) showed that the acupuncture group was significantly superior to the sham group, while the other (12) reported no significant difference between these two groups. Furthermore, it was reported that acupuncture was superior to sham acupuncture in NDI (15).

#### Acupuncture vs. Conventional Medication

Nine studies (11–14, 16–20) compared the effects of acupuncture with CM. Symptom score was reported in six studies (11–14, 16, 19), and the results of four studies (11, 13, 16, 19) revealed that acupuncture was superior to CM, while others (12, 14) showed that acupuncture was equivalent to CM. An effective rate was reported in eight studies (11–13, 16–20), and the results of five studies (13, 16, 18–20) revealed that acupuncture was superior to CM, while others (11, 12, 17) showed that acupuncture was equivalent to CM. The MF-EGG was reported in one study (11), and the pooled results showed that acupuncture was equivalent to CM. PML was reported in four studies (11–14), and the results of one study (13) revealed that acupuncture was superior to CM, while others (11, 12, 14) showed that acupuncture was equivalent to CM. Two studies reported the GHET (11, 12), and the results showed that the therapeutic effect of acupuncture group was comparable to that of CM. In addition, it was reported that acupuncture treatment was superior to CM in NDI (19).

## Discussion

Systematic reviews/meta-analyses, as the highest level of evidence source in evidence-based medicine, have become an important basis for evidence-based decision-making (21). However, SRs/MAs with low quality might affect the applicability of evidence. In this study, we performed an umbrella review to collate, evaluate, and synthesize the current evidence on acupuncture for FD.

### Main Results

First, 10 SRs/MAs published between 2014 and 2021 were included, nine of which were published over the past 5 years, indicating an increasing interest in acupuncture for FD. Second, based on the AMSTAR-2 tool, the PRISMA statement, and the GRADE system, the methodological quality, reporting quality, and evidence quality, respectively, of the included studies did not meet the requirements of the Cochrane Handbook, which means that the conclusions of the included studies may be different from the real situation and cannot provide reliable evidence for the investigators. Third, there is considerable room for improving the methodological quality and reporting the quality of SRs/MAs. This study highlights common areas for improvement such as protocols designed and registered in advance, comprehensive literature search strategies, providing a transparent list of excluded trials, and declaring funding and conflicts of interest. Fourth, there is considerable room for improving the methodological quality of RCTs. This study highlights the common areas for improvement in RCTs, such as a strict implementation of randomization and allocation concealment and adequate reporting of blinding methods. In addition, while almost all outcome measures lead to positive conclusions, it is worth noting that most authors were reluctant to draw definitive conclusions on the effects of acupuncture for FD due to the small size or low quality of the RCTs.

### Impact on Further Research

This study highlights several challenges for producers of SRs/MAs that should be addressed. First, by using the AMSTAR-2 tool and the PRISMA statement, it was observed that the registration of protocols was far from being appreciated by authors of SRs/MAs. Only one Cochrane SR/MA provided a protocol registration, whereas none of the other studies provided that. It is well-known that a preregistration protocol helps to improve transparency, minimize the potential risk of bias arising from the research process, reduce duplicate work between different researchers, and keep the study up-to-date (22). Authors are advocated to register protocols in a freely open database (PROSPERO, http://www.crd.york.ac.uk/prospero) to avoid study bias. Secondly, the risk of bias in identification and literature selection was far from being paid attention to by authors of SRs/MAs. That is, authors of SRs/MAs should provide a comprehensive search strategy and be aware that an appropriate range of databases or electronic sources of published literature should be included. In addition to the database search, other methods were considered for identifying relevant literature, such as meeting reports and trial registration platforms. Furthermore, a list of excluded trials and account for exclusion help to guarantee transparency; it can be presented by direct reference or as a supplementary file. Finally, funding sources should be fully reported as results from commercially funded research might be biased toward funders (23).

Based on the GRADE system, the risk of bias of the enrolled RCTs was the most common downgrading factor, indicating that the root cause of the reduced quality of evidence lies in the quality of the original RCTs. Only well-designed and rigorously conducted RCTs may reduce or avoid bias (24). Specific methods of randomization should be clearly described to reflect whether randomization has been successfully achieved. In addition, the information on allocation concealment and blind method should be fully reported. Due to the peculiarity of acupuncture, although blinding physicians is challenging, attempts should be made to blind patients, other caregivers, and outcome assessors to minimize the risk of bias. With the development of evidence-based acupuncture, it is hoped that researchers will continue to promote standardization and precision in the future by improving the technical operating procedures of acupuncture. Moreover, it should be realized that a large sample size and high-quality RCTs should be fundamental to high-quality evidence sources.

### Limitations

This study highlights areas of improvement in the SR/MA process that may help guide future high-quality research. However, the widely used AMSTAR-2 tool, PRISMA statement, and GRADE system are all subjective evaluation tools, and the accuracy of assessor's assessments cannot be guaranteed.

## Conclusion

Acupuncture seems to have a promising efficacy in the treatment of FD. Although the quality of the included SRs/MAs was generally low and defects were frequent, our study highlights areas where methodologies need to be improved.

## Data Availability Statement

The original contributions presented in the study are included in the article, further inquiries can be directed to the corresponding authors.

## Author Contributions

JH and JL initiated the study design and drafted the manuscript. ML, JingM, ZL, and JinxM helped with the implementation of this work. FW and XT contributed to methodology, review, and editing. All authors read and approved the final manuscript.

## Funding

This study was supported by the National Natural Science Foundation of China [Nos. 81873297 and 81804078], the State Administration of Traditional Chinese Medicine Digestive Refractory Disease Inheritance and Innovation Team Project [No. ZYYCXTD-C-202010], and the Excellent Young Scientist Fund of China Academy of Chinese Medical Sciences [No. ZZ13-YQ-006].

## Conflict of Interest

The authors declare that the research was conducted in the absence of any commercial or financial relationships that could be construed as a potential conflict of interest.

## Publisher's Note

All claims expressed in this article are solely those of the authors and do not necessarily represent those of their affiliated organizations, or those of the publisher, the editors and the reviewers. Any product that may be evaluated in this article, or claim that may be made by its manufacturer, is not guaranteed or endorsed by the publisher.
